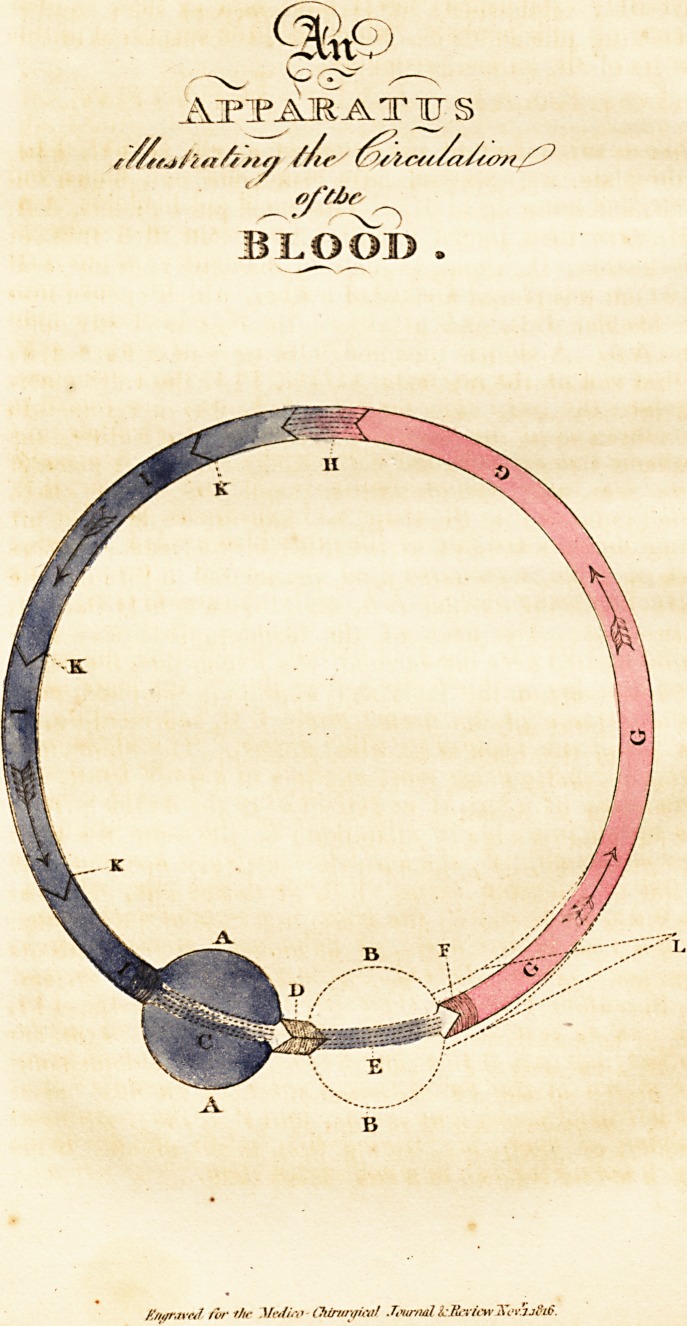# Dr. Johnson's Reply to the Foregoing Paper

**Published:** 1816-11

**Authors:** James Johnson

**Affiliations:** St. George's Square, Portsea, October 1, 1816


C. H. PARRY.

---

### Dr. Johnson's *Reply to the foregoing Paper.*

To C. H. Parry, M. D. &c.

Sir,

The perusal of the foregoing paper not having removed all doubts from my mind, I happened to devise an apparatus, which, I trust, however simple it may appear

An

APPARATUS

*illustrating the Circulation of the*

**BLOOD.**

*Engraved for the Medico-Chirurgical Journal & Review Novr. 1. 1816.*

will elucidate the subject more than many pages of argument. By means of this apparatus, I think your principal position, the non-dilatation and non-contraction of arteries during the systole and diastole of the heart, will be incontrovertibly established; but I hope also to shew another interesting phænomenon, which is little suspected in this process of the animal œconomy.

---

Six or seven feet of the ileum of a calf, G G G, I I I, in the plate, were washed clean inside and out, blown full of air, and hung up to dry. Two small pig bladders, A A, B B, were then joined together by means of a tube of sole leather, D, about an inch in diameter; at one end of which was placed a valve of leather, which opened into the bladder B B, and prevented the regress of any fluid into A A. A similar tube and valve were next fixed at F, in that end of the intestine, G G G, I I I, the valve opening into the gut. The bladder, A A, was now joined to the other end of the intestine, by means of a leather tube the same size as at F, but without any valve. A piece of twine was now wound tightly round the bladder B B, which reduced it to the shape and appearance E. The air being now pressed out of the other bladder and intestine with the hand, a coloured fluid was poured in through the valve F, till the bladder A A, and the tube G G G, I I I, were filled. The neck of the bladder, B B, was then firmly secured over the valvular tube F, and then the whole apparatus lay on the table as it appears in the plate, with the exception of the dotted circle B B, representing, in the plate, the bladder E, when dilated. The whole now formed a pretty exact representation of a single heart, the circulation of which is as perfect as in the double. A A, the auricle in a state of dilatation; C, the same in a state of contraction. D, the auriculo-ventricular opening, and mitral or tricuspid valve. B B, or dotted line, the ventricle when dilated; E, the same, in a state of contraction. F, the root of the aorta, or pulmonary artery, with its semilunar valves. G G G, the arterial canal or system. H, the capillary system (hereafter to be described). I I I, the venous system. K K K, valves, hereafter to be described, but not at this time fixed. Before commencing the march of the circulation, I squeezed the fluid out of the left-hand bladder, or auricle, into B B, the right-hand bladder, or ventricle; having first taken off the twine which bound it down in a contracted state.

*Experiment* 1st.—The auricle was now contracted as at C, and grasped by my left hand. The ventricle, B B, was full, and dilated to the dotted line. The heart was now in a state of ventricular diastole. Some bubbles of air, which were unavoidably included while joining the ventricle to the aorta, floated on the upper part of the bladder B B. My right hand now contracted on the ventricle, and relaxed from the auricle, in imitation of the action of the heart. Instantly every drop of the fluid round the whole circle was in simultaneous motion. The valve D we could see firmly shut; the fluid was gushing through the valve F, whirling round the circle, rushing into the bladder A A, and raising it up under my left hand, which I relaxed at the same time. During this systole of the ventricle, the air-bubbles were dancing round the upper segment of the tube, and indicated the march and velocity of the fluid with great exactness. By the time that the ventricle bladder, B B, was contracted, the auricle bladder was filled; I immediately pressed the latter, and relaxed my grasp of the former. That instant every globule of water and air throughout the whole tube became fixed, as if set in adamant; the valve F was seen close shut, and the liquid boiled through the valve D, till the auricle bladder was empty, and the ventricle filled: then changing the pressure from the auricle to the ventricle, every particle of fluid throughout the entire circle was again in instantaneous and simultaneous motion. These alternate dilatations and contractions of the bladders were carried on for some time with the same result. At each systole of the ventricle bladder, a vibratory motion was perceived along the circling tube, but no dilatation nor contraction, except when I happened not to relax my left hand properly on the auricle; then a dilatation of the tube was visible, from the impeded reception of the fluid into the auricle. A finger, placed on the tube during the systole of the ventricle bladder, felt a distinct pulsation, precisely similar to that of the pulse in a living artery. But, during the diastole, nothing could, of course, be felt, because the fluid was at rest. Occasionally, during the interval between one pulsation and another, a kind of faint motion in the tube was both seen and felt, and the air-bubbles at those times made a small retrograde movement in all parts of the tube. This we soon discovered to be occasioned by the contraction of the auricle, whereby a pressure was made against that end of the column of fluid which corresponds with the cava, and which reaction, in fact, closed the valve F, at the other end of the column, during the diastole of the ventricle. The harder we pressed upon the bladders in the alternate order above described, the more rapidly the fluid whirled round the tube during the systole of the ventricle, and the more quickly it passed from one bladder to the other in the diastole. But with whatever velocity it ran in the *former*, the air-bubbles decisively proved that it never advanced an iota during the *latter*; but, on the contrary, very generally made something like a minute recoil, till the valve F was closed.

*Observations.* I presume that the above will be considered as a fair imitation of the circulation in a bilocular heart and vascular system. The tube neither dilated nor contracted, when the heart or bladders were properly worked; yet, at each systole, it communicated to the finger the distinct sensation of a pulse. So far, Sir, the experiment confirms your position, that the arteries have, in common circumstances, no dilatation, nor contraction; but I think, Sir, your sagacity has already discovered, that your other position, namely, that "*it seems to you that the fluid must continue to move for a certain time after the power which has impelled it has ceased to act, just as an arrow continues to fly after it has left the bow,*" is completely overturned. The experiment is open to you, Sir, and you will find that the moment the ventricle ceases to contract, though pressed during that contraction with the force of a giant, that instant the column of fluid in the tube becomes as quiescent as if it were converted into ice. How is it possible to be otherwise in "*the case before us, which is that of a full tube, forming a circle, closed at both ends?*" When, as in the diastole of the ventricle, the fluid has ceased to enter at one end of the tube G, and depart from the other at I, how is it possible to move, unless it dilate that tube, or become itself condensed into a less space? The former, you deny; the latter is known to be impracticable. Your comparison of the blood rushing through the artery to an arrow flying through the air, is, in my opinion, not very a propos. The arrow divides and separates the air, which closes behind it; the blood does *not* separate the sides of the artery farther than before, nor will you allow that they close upon it behind.

I think your simile of the "series of billiard balls in close contact with each other" is more to the purpose; and by this I am ready to be judged. We will suppose a chain of these balls to extend from the valve D round through the ventricle BB and tube GGG, I I I, to the entrance of the auricle, which we shall leave *empty,* as one chamber of the heart must always be. We will suppose, for the sake of argument, that the ventricle BB holds six balls, the tube holding only a single series. The ventricle now contracts, and throws the six balls, in series, through the valve F. A single glance at the apparatus will convince us, that, unless the tube give out in some part, and allow the balls to ride, the *entrance* of each ball into the origin of the tube GGG must be precisely synchronous with the exit of one from the termination of the tube III; and consequently, that the final *depletion* of the ventricle BB will be exac y synchronous with the final *repletion* of the auricle AA. Then comes the next operation. The auricle AA contracts and throws the balls through the valve DD into the ventricle BB, which will be completely filled only when the auricle is completely emptied. But, in what state was the chain of balls *round the whole tube,* during this last operation? Most indubitably *quiescent.* The denial of a swell in the arterial tube during ventricular systole is fatal to your position of a *continuance* of motion during ventricular diastole:—The admission of such a swell is fatal to your position of non-contraction and non-dilatation; and I shall presently shew that it would prove fatal to the machine itself.

In the 7th vol. of this Journal, page 27, I stated it as my opinion, that "it is absolutely impossible that a fluid can continue to flow through a tube, while there is no fluid entering that tube, nor contraction of the tube itself taking place, which must be the case during the diastole of the ventricle, if the arteries remain the same." I now reiterate that opinion, and the foregoing apparatus and experiment fully prove it. But I freely confess, that I was entirely wrong in adhering to the general belief of dilatation and contraction in the arteries being the cause of the pulse. From numerous experiments which I have made, and am making, I am confident that I shall confirm the truth of your position in this respect; and, in addition, prove, that in health, and under ordinary states of the system, the blood in the vessels, both arterial and venous, is perfectly quiescent during the diastole of the ventricles, and in motion only during their systole. A doctrine so entirely repugnant to general opinion, and to what we are taught in the schools of anatomy and physiology, will at first be disbelieved, as was the Harveian doctrine itself; but,

"Omnia vincit veritas et prevalebit."

The apparatus which elucidates this subject is so simple that it will be in the hands of many; and certain I am, that it will bring conviction to the most sceptical mind. But some difficulties must be cleared up. From *a priori* reasoning, there is nothing unnatural or unreasonable in these alternations of motion and quiescence in the circulation. There is hardly any thing in Nature which is not subject to alternations. The seasons; day and night; waking and sleeping; exercise and repose; eating and abstinence, inspiration and expiration, &c. &c. all alternate; so that to conceive the idea of any thing constant is almost unnatural. In respect to the motion of the blood, when we recollect that at one, and by far the most important, point of the circle—the ventricle of the heart, a pause in the current unequivocally alternates with a rush of the fluid, what is there unreasonable in the supposition that the same should prevail throughout? But, it is said, if we cut an artery, the blood will flow in the diastole of the ventricle. If we cut a leaden pipe in the street, when the water is perfectly quiescent, the fluid will gush out many feet in height. Your experiments, Sir, have proved, that in ordinary states of the circulation, the calibres of the arterial trunks are greater than they would assume, were they not distended with blood; hence, it is evident, that they will continue to pour out their contents during systole and diastole, through any artificial opening, till their elastic and muscular coats can no longer contract; —this elasticity and contractility of the arteries producing precisely the same effect as the pressure of water in the cistern produces on the leaden pipe that leads from it.

But the blood is to be forced through the capillary system; and for this purpose, it is supposed the arteries must assist the heart. It has been calculated by Keil, that the bore of the aorta is, to that of the extreme vessels united, as 1 to 44.500. Although this may be wrong, it is allowed by all, that the united calibres of the branches infinitely exceed that of the trunk:—What, then, can occasion the great difficulty of transmission? It would, surely, be more reasonable, to expect difficulty in the *venous* return of the blood from such numerous bores through, at last, a single trunk.

But when we reflect that the blood is an incompressible fluid, extending in a column or columns from the root of the aorta round to the root of the cava, we shall see that every point of that column, or series of columns, sustains itself against the pressure of the circumvesting pipe, and that the fluid can only attempt to escape from this pressure through one or both ends of the tube. Now, during the diastole of the ventricle, the semi-lunar valves check its exit from the column at one end, the contraction of the auricle prevents its exit from the other end. It must, of necessity, therefore, either remain quiescent, or diminish in one part of the circle, and accumulate in the other. If more be thrown into the aorta, at each systole, than is discharged from its capillary terminations, then a swell is inevitable: on the other hand, if more rush into the auricle from the cava during the above-mentioned systole, than is received from the arterial terminations in the same period, a diminution of the venous trunks must be the unavoidable consequence. In every way that we view the affair then, the continuation of motion in either arterial or venous system during the period that the blood is passing from the auricle to the ventricle, (at which time no fluid is pouring into the one, nor out of the other system) can only be accomplished by an alternate rise and depression in the two systems.*

*Experiment* 2d. Having pierced a circular piece of cork, the size of the tube in thickness, with small holes, and fixed it at H; I found that by pouring water in at F, the capillary septum did not transmit the fluid so quickly as the valved tube F. The apparatus was then put in order, and the circulation commenced. But here a new train of phænomena took place. At each contraction of the ventricle B B, the arterial side of the tube G G G swelled, and throbbed violently, while the venous side of the tube I I I remained perfectly motionless; and now I found that the harmony between the auricle and ventricle was destroyed. When the ventricle was contracted, the auricle was not above two-thirds dilated; the remaining third was some time in filling, and did not become perfectly full in any reasonable time. During the systole of the ventricle, about two-thirds of the quantity held by the ventricle, passed through the capillary tubes at H. The remaining third slowly passed through by the recoil of the tube and the laws of gravitation, till which time the auricle did not fill. When I put the auricle [two-thirds full] in motion, as

---

* If I have avoided any allusion to the principle lately maintained by Dr. Carson of *active* dilatation in the chambers of the heart, it is not because I deny it, but because it in no way affects, either pro or con, the positions which I am endeavouring to establish.

soon as the ventricle was empty, the fluid entering it from the venous tube I I I being checked, while the remaining third of the ventricle full was running in at H, a dilatation and throb were clearly perceptible throughout the venous side of the tube, but not so strong as previously in the other, because the dilated tube G G G did not contract with such force as I had applied to the bladder B B before.

*Observations.* This experiment shews the effects of a dilatation of the arterial system, during the systole of the ventricle. The arteries giving way, and dilating before the force of the heart, prevent the latter from pushing the contents of its ventricular chamber through the capillary system at once; the consequence of which is, that its auricular chamber must wait till the arteries have supplied the deficiency; but what is worse, the strong impulse of the ventricle being lost before the auricle is filled, the latter is not properly filled at all, and thus the harmony of action between the two chambers is destroyed. But let us examine more nearly the absurdity of this supposed mode of circulation. Those who maintain that during each systole of the ventricle a portion of a ventricle full of blood is pushed through the capillary system, the remainder being left in the dilated arteries to follow, must allow, that the ventricle here evinces more power than the whole arterial and capillary systems together. It drives a portion of its blood through the one system, while it dilates the other with the remainder. Thus the force of the stronger body (on this principle) is squandered away, in consequence of the yielding of the weaker, the arteries.

But supposing that a dilatation of the arterial tube G G G, similar to the dotted line L, should take place during each systole of the vèntricle B B, to feed the supposed stream through the capillaries H, during the diastole of the ventricle :—It follows, that blood continues to run into the venous system at the capillary end, during the contraction of the auricle, [corresponding with the diastole of the ventricle] when none is disgorging from the auricular end. What must be the consequence of this? Why, a swell, a dilatation, or pulse in the veins during the diastole of the ventricle, precisely what happened when the arterial side of the tube G G G was dilated by the contracting bladder B B, and a consequent stream was kept up through the capillary tubes at H, while the auricle A A was pushing its contents into the ventricle.

Having, now, Sir, I hope fully shewn, both from experiment and the laws of hydrostatics, that dilatation and contraction in the arterial system must inevitably produce dilatation and contraction in the venous, proportioned to the difference of calibre in those two systems, I shall next proceed to prove that such alternate motions in either or both systems, are incompatible with the due performance of those important functions which they are destined to carry on.

As all the blood which continues to pass through the capillaries into the venous system, during ventricular dilatation and auricular contraction, when one end of the venous system is receiving and the other *not* discharging, must accumulate in the veins, let us next enquire how it is to be disposed of afterwards. When the contrary state succeeds, namely, while the ventricle is contracting and the auricle is dilating, a fuller and stronger current of blood is rushing through the capillaries into the veins, impelled by the great power of the heart.—The auricular end of the venous system opens, indeed, but it can hardly be supposed that it disgorges more into the auricle than it receives from the arteries, unless we assign to the venous system a power of counteracting and overcoming the force of that chamber of the heart which is, on this principle, capable of dilating the arterial system, and pouring a torrent of blood through the capillaries at the same time. And by what means can the veins contract at this period of distention—seeing that they possess no muscularity and little elasticity? The thing is impossible and aburd.

Since, then, what passes into the venous system during the diastole of the ventricles, cannot be disgorged during that diastole, nor during the subsequent systole, it follows, that an accumulation must ensue till the whole blood is in the veins, or till the veins cease to dilate, when dilatation and contraction of the arteries must also cease, and then the blood will be alternately in motion and quiescence, as I am endeavouring to prove.

If it be said that muscular action will clear the venous system of any superabundance of blood, I answer, that the muscular action must then be *always* necessary, and what shall become of us during sleep? If it be asked, why the blood does not flow *per saltum* from a vein when opened in venæsection; I answer, that the ligature above forms an artificial reservoir, from which the blood of course issues in an even stream; and where the blood continues to flow after the ligature is removed, it is because the orifice presents a freer vent than the subcutaneous vein along the higher part of the arm, which comes to the same thing.

Besides, in each individual branch of a vein the blood is running slower, by one half at least, than in the artery, and is flowing from a smaller into a larger bore :—The inosculations also are infinitely more numerous; and hence there is neither the *per saltum* escape when the vessel is cut, nor the sensation of pulse when it is compressed.

It has been suggested to me, that a continued stream of blood may be carried on through the capillaries in the same manner as air through the nozzle of a pair of double bellows. The analogy seems at first sight somewhat feasible, but, on closer inspection, the illusion vanishes. In the first place there must here be dilatation of the aorta or its branches, similar to the dilatation of the upper bellows during the contraction of the lower, and, *vice versa*. Your experiments, Sir, have set the question of dilatation and contraction at rest. But granting that there was a dilatation of the root of the aorta in imitation of the bellows, let us see how the *latter* would go on, if placed in similar circumstances with the former. If a hose were led round from the nozzle of the bellows to a reservoir under the valve of the lower bellows, and this hose was made *dilatable,* like the venous system, but without disposition to contract; and if the same air must pass round and round without compressibility, then the simile will stand good. The upper bellows will represent the arterial system, being capable of dilatation and contraction : The lower bellows will represent the ventricle or grand mover; the reservoir or bag underneath for the reception of the returning air, will represent the auricle; the hose, the veins; the nozzle, the capillary system. We will suppose the upper bellows to be two-thirds filled for the arteries, the lower quite distended; the veins two-thirds filled; the reservoir empty, and ready to receive the returning current. The lower bellows now contracts strongly, forcing three-fourths of its contents through the nozzle, the other fourth through the valve into the upper bellows, dilating that division of the machine which is to feed the fire at the nozzle while the lower division is replenishing. At this instant, however, the reservoir is not filled by the one-fourth which is in the upper bellows. What then must be done? If we wait till the full quantity comes round, a pause, however short, must take place in the stream at the nozzle, before another blast can be impelled, and then the argument for perpetual motion is at an end. We do not therefore wait. The moment the lower bellows, or ventricle, is empty, we squeeze into it the three-fourths of air from the reservoir, and dilate it though imperfectly. While we are doing this, the upper bellows is impelling a diminished current through the pipe and into the hose. But it cannot pass along the hose as it enters, because the reservoir at the other end is contracting and pushing its contents into the lower bellows. The air, therefore, remains in the hose, and distends it a little. The lower bellows now contracting, again drives three-fourths of its *diminished* load through the nozzle, leaving a fourth, as usual, in the upper bellows for feeding the flame. During this operation the hose cannot contract to its original dimensions, even had it a contractile power, because three-fourths of the power of the lower or main bellows are exerted in driving the air round into the reservoir through the hose. Hence, at each dilatation of the lower, and contraction of the upper bellows, an accumulation takes place in the dilatable hose, till the whole air is confined there, and the machine stops! The analogy then is fallacious; and why? Because the lower bellows receives its full quantum from an open unlimited atmosphere, instead of being confined to the same current which it expels.

I trust, Sir, that I have now established the following points. 1st. The truth of your position, that in ordinary states of the system, there is neither dilatation nor contraction in the arteries corresponding with the systole and diastole of the ventricles. 2d. That during auricular contraction and ventricular dilatation, the blood is quiescent both in the arterial and venous systems. 3d. That were there dilatation and contraction in the arteries corresponding with systole and diastole; the same movements, though in a less perceptible degree, must inevitably take place in the veins. 4th. That were the arteries to dilate during the systole of the ventricles, so as to keep up a diminished current through themselves and the capillaries during the diastole, then an accumulation would take place in the veins that would ultimately destroy the harmony between the two systems, and even the machine itself.

These positions I am prepared by experiments and arguments to support; and I am inclined to believe, Sir, that this view of the circulation will lead to the explanation of many phænomena, hitherto involved in obscurity, and that it will even bear upon *practical points,* connected with derangements in the circulating organs, and elucidate the effects and symptoms of these derangements.

On a subject so interesting to science and humanity, you have already, Sir, thrown great light; and although in establishing the facts of non-contraction and non-dilatation, you seemed to forget the *consequences* of these—

*alternate motion and quiescence in the blood*, yet, I hope, on a re-consideration of the subject, you will acknowledge the correctness of the reasonings and deductions in this paper, and pursue the investigation with that ability which is so conspicuous in every part of your writings.

I am, Sir,
With the most profound respect,
Your most obedient servant,
JAMES JOHNSON.

St. George's Square, Portsea,
October 1, 1816.

☞ Some further experiments and improvements in the construction of the Apparatus will be detailed in a future Number.

## Figures and Tables

**Figure f1:**